# Macrophage Migration Inhibitory Factor Inhibits the Migration of Cartilage End Plate-Derived Stem Cells by Reacting with CD74

**DOI:** 10.1371/journal.pone.0043984

**Published:** 2012-08-27

**Authors:** Cheng-jie Xiong, Bo Huang, Yue Zhou, Yan-ping Cun, Lan-tao Liu, Jian Wang, Chang-qing Li, Yong Pan, Hai Wang

**Affiliations:** 1 Department of Orthopedics, Xinqiao Hospital, Third Millitary Medical University, Chongqing, People’s Repulic of China; 2 Cancer Centre, Daping Hospital and Research Institute of Surgery, Third Military Medical University, Chongqing, People’s Republic of China; University of Medicine and Dentistry of New Jersey, United States of America

## Abstract

**Background:**

Macrophage migration inhibitory factor (MIF) is a multifunctional cytokine that regulates inflammatory reactions and the pathophysiology of many inflammatory diseases. Intervertebral disc (IVD) degeneration is characterized by an inflammatory reaction, but the potential role of MIF in IVD degeneration has not been determined. Recent studies have shown that MIF and its receptor, CD74, are involved in regulating the migration of human mesenchymal stem cells (MSCs); Thus, MIF might impair the ability of mesenchymal stem cells (MSCs) to home to injured tissues. Our previous studies indicated that cartilage endplate (CEP)-derived stem cells (CESCs) as a type of MSCs exist in human degenerate IVDs. Here, we investigate the role of MIF in regulating the migration of CESCs.

**Methods and Findings:**

CESCs were isolated and identified. We have shown that MIF was distributed in human degenerate IVD tissues and was subject to regulation by the pro-inflammatory cytokine TNF-α. Furthermore, *in vitro* cell migration assays revealed that nucleus pulposus (NP) cells inhibited the migration of CESCs in a number-dependent manner, and ELISA assays revealed that the amount of MIF in conditioned medium (CM) was significantly increased as a function of increasing cell number. Additionally, recombinant human MIF (r-MIF) inhibited the migration of CESCs in a dose-dependent manner. CESCs migration was restored when an antagonist of MIF, (S, R)-3(4-hydroxyphenyl)-4, 5-dihydro-5-isoxazole acetic acid methyl ester (ISO-1), was added. Finally, a CD74 activating antibody (CD74Ab) was used to examine the effect of CD74 on CESCs motility and inhibited the migration of CESCs in a dose-dependent manner.

**Conclusions:**

We have identified and characterized a novel regulatory mechanism governing cell migration during IVD degeneration. The results will benefit understanding of another possible mechanism for IVD degeneration, and might provide a new method to repair degenerate IVD by enhancing CESCs migration to degenerated NP tissues to exert their regenerative effects.

## Introduction

Macrophage migration inhibitory factor (MIF) was first described as a soluble factor that is released by activated T-lymphocytes in 1966. MIF has been reported to inhibit the random migration of monocytes and macrophages [1]. Subsequently, significant quantities of MIF were found within the pituitary gland and monocytes/macrophages besides T-lymphocytes [1]–[3]. As an important proinflammatory cytokine, MIF might counter-regulate glucocorticoid effects by activating immune/inflammatory cells and promoting the expression of matrix metalloproteinases, nitric oxide and prostaglandin E2 release [1]–[3], or the release of proinflammatory and inflammatory cytokines [4], such as TNF-α, IL- 1β, IL-2, IL-6, IL-8, IFN-γ. Moreover, each of those proinflammatory and inflammatory cytokines are involved in the pathogenesis of intervertebral disc (IVD) degeneration [5]–[9]. However, a potential role for MIF in the pathogenesis of IVD degeneration has not yet been investigated.

Mesenchymal stem cells (MSCs) hold promise for use in regenerative medicine in the treatment of degenerative diseases, such as IVD degeneration [10], [11]. The therapeutic application of MSCs exploits the ability of MSCs to home to injured or degenerated tissues and facilitate the healing process [12]. The migration of MSCs is regulated by a variety of cytokines, such as fibroblast growth factor-2 (FGF-2) [13], platelet-derived growth factor (PDGF) [13] and MIF [14]–[16]. FGF-2 and PDGF can facilitate the migration of MSCs to sites of injury; conversely, MIF inhibits MSCs migration into the sites of inflammation [13]–[16]. Elevated levels of MIF in injured tissues could interfere with therapeutic effects of MSCs [14]–[16]. The MIF antagonist, ISO-1, inhibits the biological function of MIF and enhances the migration of MSCs. ISO-1 has the potential to exert a therapeutic effect by countering the MIF-mediated inhibition of MSC migration [14]–[17].

**Figure 1:**
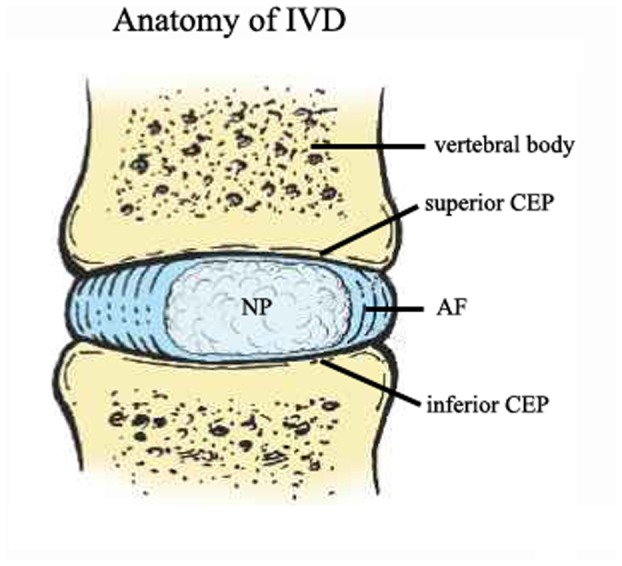
The structure of anatomy of the IVD. The IVD is composed of three distinct regions: an outer tough, collagenous AF surrounding a central highly hydrated, gelatinous gelatinous NP, between bflatter, less malleable CEPs, superiorly and inferiorly against adjacent vertebral axial surfaces.

There is an intricate and functionally sophisticated relationship between the major anatomical components of the IVD. There is a gelatinous structure located centrally, named the nucleus pulposus (NP) which is embedded concentrically within the cylindrical annulus fibrosus (AF). This pair of structures is flanked by flatter, less malleable structures, named cartilage endplates (CEP), superiorly and inferiorly against adjacent vertebral axial surfaces (Fig. 1). Normal IVD functions as shock absorbers, which transmit and distribute large loads on the spine while providing flexibility. IVD degeneration or injury leads to dysfunction and painful symptoms. Buckwalter JA believes that the lumbar spine degeneration initially occurs within then central NP of the IVD [18]. IVD degeneration is associated with a decrease in disc cell number, a loss of proteoglycan and water content in the NP [19]. However, Roberts S. et al have shown that CEP has play a crucial role in the IVD degeneration. The lesion of the transportation function of CEP accelerates the loss of proteoglycans from the NP, and IVD degeneration process [20].

Kim et al demonstrated that rabbit chondrocytes from the cartilage end plate (CEP) could migrate into the nucleus pulposus (NP) and changed a notochordal NP into a fibrocartilaginous NP by depositing fibrocartilage lamellas and fibers in a centripetal direction [21]. In their study, *in vitro* cell migration assays indicated that notochordal cells could stimulate the migration of CEP chondrocytes [22], [23]. However, whether chondrocytes can also migrate in human degenerated CEP and the specific molecular mechanism underlying cell migration have not yet been determined. Conversely, NP cells can be stimulated to produce a variety of proinflammatory and inflammatory cytokines during IVD degeneration [24], [25], and whether these cytokines are involved in regulating migration should be considered.

Our group recently demonstrated that stem cells exist in human degenerated CEP and that cartilage endplate-derived stem cells (CESCs) are superior to BM-MSCs in terms of osteogenesis and chondrogenesis [26], [27]. In this study, we hypothesized that CESCs exert the function of MSCs by homing to degenerated NP tissues during IVD degeneration and that NP cells could release MIF to inhibit CESCs migration via interaction with the CD74 receptor.

## Materials and Methods

### Ethics Statement

The IVD tissues used in this study was obtained from seven patients who underwent posterior discectomy and fusion for lumbar degenerative disease (Table 1). The tissues, life information, or gene information from patients were necessary in this study. The Ethics Committee of Xinqiao Hospital, Third Military Medical University, specifically approved this study and institutional safety and ethical guidelines were followed. Written informed consent was obtained from each patient, and extensive precautions were taken to preserve the privacy of the participants donating tissue. All the procedures were in accordance with the Helsinki Declaration.

**Table 1:** Details of discs applied for stem/progenitor cells isolation.

Procure	disc level	Modified Piffirman grade	Gender	Age
lumbar disc herniation	L4-L5	Grade5	F	63
lumbar disc herniation	L4-L5	Grade6	M	69
lumbar disc herniation	L4-L5	Grade4	M	61
lumbar disc degeneration	L4-L5	Grade3	M	42
lumbar disc degeneration	L5-S1	Grade3	M	38
lumbar disc degeneration	L5-S1	Grade4	F	37

### Tissue Procurement

With approval from the institutional review board of the Xinqiao Hospital, and specific (documented) informed consent in each case, degenerated human disc tissue was obtained from patients who underwent posterior discectomy and a fusion procedure for lumber degenerative disease. A modified Piffirman grading system [28] for lumbar disc degeneration was employed in this study. Table 1 provided detailed information about the subjects. Degenerated NP and CEP were separated by two experienced operating surgeons according to a protocol described in the literature [29] and were washed, to remove blood, with sterile 0.1 M phosphate buffered saline (PBS, pH 7.4).

### Cell Culture and Stimulation

Cells from NP were obtained via a previously described protocol [26], [27]. Briefly, The NP tissues were carefully examined to remove any obvious granulation tissue or ligament tissue, and then were minced into 1 mm^3^ blocks. Cells were isolated by digestion medium containing Dulbecco’s Minimum Essential Medium (DMEM)/F12, 0.2% type II collagenase (Sigma-Aldrich, USA) and 1% penicillin-streptomycin (Invitrogen Gibco, USA) at 37°C for 12 hours. The suspended cells were filtered through a 70-µm cell filter to minimize cell aggregates. Then the cell suspension was transferred to a sterile 15 ml polypropylene culture tubes and centrifuged for 5 minutes at 100×g. The suspension solution was discarded, pellets resuspended in the expansion culture medium containing DMEM/F12, 10% fetal calf serum (FCS) and 1% penicillin-streptomycin (Invitrogen Gibco, USA). Total cell number per tissue specimens was 1.5×10^4^ to 5.0×10^4^. NP cells were collected and plated in culture bottles, and then cultured in the expansion culture medium under a humidified atmosphere containing 5% CO2 at 37°C. The medium in the wells was replaced with fresh medium twice a week and subcultures were performed when the cells were about 90% confluent. To eliminate contamination with macrophages or other leukocytes, cells were subcultured 3 times.

For the enzyme-linked immunosorbent assay (ELISA), NP-cells were plated in 24-well plates in Dulbecco’s Minimum Essential Medium (DMEM)/F12, supplemented with 10% fetal calf serum (FCS) and 1% penicillin-streptomycin (Invitrogen Gibco, USA). The medium in each well was replaced with fresh medium once per week. When the cells were approximately 90% confluent, the culture medium was replaced with serum-free medium and then with serum-free medium containing various concentrations of TNF-α (0–100 ng/ml; R&D Systems, Minneapolis, MN,USA ). After 12 hours, the supernatants were collected for MIF ELISA [30]. For reverse transcription (RT)–PCR studies, the cells were collected for mRNA analysis after 6 hours.

### Flow Cytometry

CESCs were obtained via a previously described protocol [26], [27] and then subjected to flow cytometry analysis for quantitation of multipotent MSC receptor expression [31]. Briefly, NP, AF and subchondral bone tissues around blocks of CEP were removed using ophthalmic operating set under dissecting microscope (4×magnification), and then CEP cells were isolated as cells from NP. 2% low-melting point agarose (Invitrogen, USA) was sterilized by autoclaving and then equilibrated to 37°C before the next procedure. Culture dishes (60 mm in diameter, Costar Corning, USA) were coated with 1% low-melting point agarose which was mixed with an equal volume of 37°C 2×DMEM/F12 and 2% low-melting point agarose and the excess agarose was removed by aspiration. Subsequently, a mixture of 0.75 ml of DMEM/F12, 0.75 ml of 2% low-melting point agarose and 1.5 ml of 20% FCS DMEM/F12 containing 5×10^4^ P1 CEP cells was added to the culture dishes. The final concentration of FCS was 10%. Culture dishes were held at 4°C for 15 minutes until the gels solidified. The culture dishes were then incubated in a humidified atmosphere containing 5% CO2 at 37°C. Culture medium were Changed with DMEM/F12 supplemented with 10% FCS and 5 units/ml penicillin and streptomycin twice a week. After 6 weeks, cell clusters (diameter greater than 50 mm) were isolated using a sterile Pasteur pipette and subcultured in multi-well plates (Costar Corning, USA). Passage 3 CESCs were used in the study.

The cells were trypsinized and the cell suspension was stained with phycoerythrin (PE) or fluorescein isothiocyanate (FITC) or allophycocyanin (APC) -conjugated antibodies: CD73-FITC, CD90-FITC, CD105-PE, CD14-FITC, CD19-APC, CD34-FITC, and CD45-FITC. IgG was used as an isotype control. All antibodies were purchased from eBioscience(USA). The final antibody concentration was at 1 µg/200 µl. After incubating for 30 minutes 37°C, the cells were washed three times with PBS. Finally, Labeled cells were subjected to single channel flow cytometry analyzer (BD, USA) and the percentage of stained cells was calculated relative to isotype control.

For CD74, the cells were trypsinized and washed three times with PBS containing 10% FCS and 1% sodium azide and then incubated with a primary antibody rabbit anti-human CD74 antibody (1∶100, Epitomics, CA, USA) in PBS containing 3% bovine serum albumin (BSA) or control lgG for 2 hours. The cells were washed three times with PBS and then incubated with a FITC-conjugated goat anti-rabbit IgG secondary antibody (1∶100, ZSGB-BIO, Beijing, China) in PBS containing 3% BSA for 30 minutes at room temperature in the dark before three PBS containing 3% BSA and 1% sodium azide washes. Finally, Labeled cells were subjected to single channel flow cytometry analyzer (BD, USA) and the percentage of stained cells was calculated relative to isotype control.

### Multilineage Differentiation Potential in vitro

To analyze osteogenic differentiation, CESCs were plated at a concentration of 2×10^5^ cells/well in 6-well plates. The medium in the wells was replaced with fresh expansion culture medium every 3 days in standard conditions (humidified atmosphere at 37°C with 5% CO2 ) until the cells were fully confluent. The cells were supplemented with osteogenic differentiation medium (Cyagen Biosciences Inc, USA) containing 10% FCS, 10 nM dexamethasone, 10 mM β-glycerophosphate, and 0.1 mM L-ascorbic acid-2-phosphate by replacing the medium every 3 days. After a 2–3 week induction period, and the cells were subjected to histological examination. Negative control wells were supplemented with expansion medium every 3 days. After the cells had differentiated, osteogenic differentiation medium was removed and the wells were rinsed with PBS and fixed with 2 mL of 4% formaldehyde solution for 30 minutes. The wells were washed twice with PBS, the cells were stained with 1 ml alizarin red S for 3–5 minutes. The wells were washed twice with PBS, and could now be visualized by and analyzed under microscope.

To assess adipogenic induction, CESCs were plated at a concentration of 2×10^5^ cells/well in 6-well plates. The medium in each well was replaced with fresh expansion culture medium every 3 days under standard conditions (humidified at 37°C with 5% CO2 ) until the cells were 100% confluent. The cells were supplemented with adipogenic differentiation induction medium (Cyagen Biosciences Inc, USA) containing 10 µg/mL insulin, 1 µM dexamethasone, 500 µM 3-isobutyl-1-methyl xanthine, and 100 µM indomethacin for 72 hours. Subsequently, the medium in each well was replaced with adipogenic differentiation maintenance medium (Cyagen Biosciences Inc, USA) containing 10 µg/ml insulin in DMEM and 10% FCS for 24 hours. After 3–5 induction/maintenance cycles, the cells were supplemented with adipogenic differentiation maintenance medium for an additional 7 days by replacing the medium every 3 days. After a 3–5 week induction period, the cells were subjected to histological analysis. Negative control wells were supplemented with expansion medium every 3 days. Lipid droplets were stained with Oil Red O. After the cells had differentiated, adipogenic differentiation medium were removed from the wells and the cells were rinsed with PBS, and fixed with 2 mL of 4% formaldehyde solution for 30 minutes. The wells were washed twice with PBS, the cells were stained with 1 ml oil red O working solution for 3–5 minutes. The wells were washed twice with PBS, and could now be visualized by and analyzed under microscope.

To assess chondrogenic differentiation, CESCs were centrifuged at 200 g for 5 min in a 15 ml-capacity polypropylene tube. The cell pellets were supplemented with chondrogenic differentiation medium (Cyagen Biosciences Inc, USA) containing 10 ng/ml transforming growth factor (TGF)- β3, 10^−7^ M dexamethasone, 50 µg/ml ascorbate-2-phosphate, 40 µg/ml proline, 100 µg/ml pyruvate, and 1∶100 diluted ITS+Premix, by replacing the medium every 3 days. The cells were maintained in standard conditions (humidified at 37°C with 5% CO_2_). After a 2–3 week induction period, the cells were subjected to histological analysis. Negative control groups were supplemented with expansion medium every 3 days. The pellet were fixed with formalin and embedded with paraffin. 3–4 µm paraffin sections were stained with the alcian blue solution for 30 minutes. the cells were washed in running tap water for 2 minutes, and could now be visualized by and analyzed under microscope. Blue staining indicated synthesis of proteoglycans by chondrocytes.

### Immunocytofluorescence

NP cells were plated in cell culture dishes (NEST Biotech Co., LTD, China). When confluence was reached, the cells were fixed in 100% methanol and treated with a blocking solution containing 1% bovine serum albumin and 0.1% Triton X. The cells were stained with primary polyclonal rabbit anti-human MIF antibody (1∶100, Santa Cruz, CA, USA) overnight at 4°C. The cells were then washed three times in PBS containing 0.1% Triton X and stained with a FITC -conjugated goat anti-rabbit IgG secondary antibody (1∶100, ZSGB-BIO, Beijing, China) for 1 hour. Subsequently, cell nuclei were stained with 4′,6-diamidino-2-phenylindole (DAPI; 0.1 µg/ml, Sigma, USA). For all staining procedures, negative controls were obtained via omission of primary antibody and via incubation with a blocking solution. The cell culture dishes were examined with a laser-scanning confocal microscope. Monoclonal rabbit anti-human CD74 (1∶100, Epitomics, CA, USA) was used to detect CD74 on CESCs, and immunofluorescence staining was performed according to the procedure described above.

### Immunohistochemistry of IVD Tissues

Immunohistochemistry was used to localize MIF and its receptor CD74 in 6 IVD samples (Table 1). Briefly, 4 µm paraffin sections were dewaxed and incubated with 3% hydrogen peroxide for 15 minutes. After blocking endogenous peroxidase activity, antigen retrieval was performed by heating the sections in citrate buffer. After washing three times in PBS containing 0.1% Triton X, the samples were blocked with a solution containing 1% bovine serum albumin and 0.1% Triton X. The sections were incubated with a primary polyclonal rabbit anti-human MIF antibody (1∶100, Santa Cruz, CA, USA) or a monoclonal rabbit anti-human CD74 antibody (1∶100, Epitomics, CA, USA) overnight at 4°C. The sections were washed and then incubated with a polymer peroxidase anti-rabbit IgG antibody (ZSGB-BIO, Beijing, China) for 30 minutes at room temperature. Immunoreactive proteins were revealed with 3, 3′-diaminobenzidine (DAB) and the samples were counterstained with hematoxylin. For all staining procedures, negative controls were obtained via omission of primary antibody and via incubation with a blocking solution. Images were obtained with an Olympus BX60 microscope (Olympus, Tokyo, Japan).

### Small Interfering RNA (siRNA) Transfection

NP cells were transiently transfected with a mixture of 33 nmol/liter CD74 siRNA (Santa Cruz Biotechnologies) and Lipofectamine 2000 in Opti-MEM I reduced serum medium (Invitrogen) for 48 h. Cells were then starved for 16 h in DMEM/F-12 and stimulated with MIF. Transfection efficiency was monitored using silencer GAPDH siRNA and the nontargeting silencer negative control no. 1 siRNA (Ambion Applied BioSystems, Foster City, CA). We also had optimized the siRNA transfection conditions (cell number, concentration of siRNA, and amount of transfection reagent) (data not shown).

### Western Blotting

IVD tissues were surgically obtained from 3 donors between 67 and 71 years of age (mean: 69 years), snap frozen in liquid nitrogen, and homogenized for protein analysis. The samples were resolved by SDS-PAGE in an 18% polyacrylamide gel and transferred to nitrocellulose membranes. The membranes were blocked overnight in Tris-buffered saline supplemented with Tween and 5% dry milk, incubated overnight, immunoblotted with a primary polyclonal rabbit anti-human MIF antibody (1∶1000, Santa Cruz, CA, USA), and then incubated with a horseradish peroxidase-conjugated goat anti-rabbit IgG antibody(ZSGB-BIO, Beijing, China) for 1 hour. Immunoreactive proteins were visualized via chemiluminescent detection (ECL, Amersham). Molecular weight markers were indicated on the left. Recombinant human MIF (r-MIF, R&D Systems, Minneapolis, MN, USA) was used as a positive control. The above protocol had been described by other groups [30].

### Enzyme-linked Immunosorbent Assay (ELISA)

MIF from CM was analyzed via anti-human MIF-ELISA (Westang Biotechnology, Shanghai, China) according to the manufacturer’s recommendations. A polyclonal anti-MIF antibody (Westang Biotechnology, Shanghai, China) was used as the capture antibody, and absorbance was measured at 450 nm in a microplate reader. The concentration of MIF in each sample was obtained by comparing absorbance values against the standard curve using r-MIF (Westang Biotechnology, Shanghai, China). The above protocol had been described by other groups [32].

### RT-PCR

Total RNA was extracted from macrophages using an RNeasy kit (Qiagen GmbH, Germany) according to the manufacturer’s instructions. Isolated RNA was treated with RNase-free DNase (QIAGEN GmbH) before being reverse transcribed into cDNA. The purified RNA was quantified in a spectrophotometer (Beckman, Fullerton, CA) at 260 nm and 280 nm. The RNA was transcribed into cDNA with a ThermoScriptTM RT-PCR system (Invitrogen, USA) and amplified via PCR. Amplification primers were listed in Table 2. The mRNA levels were normalized against the housekeeping gene glyceraldehydes-3- phosphate dehydrogenase (GAPDH). The above protocol had been described by other groups [30], [32].

**Table 2:** Primers used for RT-PCR analysis.

Target gene	Primer sequence
MIF	5′-GCGCGTGCGTCTGTGCC-3′
	5′-GACCACGTGCACCGCGATGTA- 3′
CD74	5′-TGACCAGCGCGACCTTATCT-3′
	5′-GAGCAGGTGCATCACATGGT-3′
GAPDH	5′-TGGGGTGATGCTGGTGCTGAGT-3′
	5′-AGGTTTCTCCAGGCGGCATGTC-3′

### Migration Assay

The migration of CESCs was evaluated with transwell cell culture chambers (8 µm pore size) (Millipore, Billerica, MA, USA) as described previously for a 24-well plate setup [14]–[16]. Briefly, CESCs at passage 3 were cultivated in serum-free DMEM/F12 for 24 hours, trypsinized, and then transferred to the upper chamber at a concentration of 2×10^4^ cells/well in 300 mL serum-free DMEM/F12. The CM from NP cells of different numbers were collected and then transferred to the lower chamber.

To test the effect of r-MIF (R&D Systems, Minneapolis, MN, USA) and the CD74-activating antibody (CD74Ab; Santa Cruz Biotechnology, CA, USA) on the CESCs, Both r-MIF and CD74Ab were added to the lower chamber containing DMEM/F12 at different concentrations. The CD74Ab were dialyzed to remove sodium azide prior to the assay. In some DMEM/F12, an antagonist of MIF, ISO-1 (Merck, Darmstadt, Germany) was added 0.5 h before MIF.

The migrated cells (on the outer bottom of transwell) were fixed in methanol and stained with 1% crystal violet; the stained cells were then counted digitally with Image Pro Plus software in 5 random fields at 200×magnification. Negative controls contained serum-free DMEM/F12.

### Small Interfering RNA (siRNA) Transfection

CESCs were transiently transfected with CD74 siRNA (Santa Cruz Biotechnologies) using Lipofectamine 2000(Invitrogen) in accordance with the manufacturer’s instructions for 48 h. Cells were then starved for 24 h in DMEM/F12 and stimulated with MIF(100 ng/ml). Samples were collected for the migration assay,and PCRs were performed with CD74 primers and reprobed with the glyceraldehyde-3-phosphate dehydrogenase (GAPDH) primers as loading controls to detect the transfection efficiency.

### Statistical Analyses

The data are expressed as the mean ±SD of three independent experiments. The data were analyzed via one-way ANOVA comparisons between factors of different groups with significance values set at P<0.05.

## Results

### In vitro Characterization of CESCs

**Figure 2:**
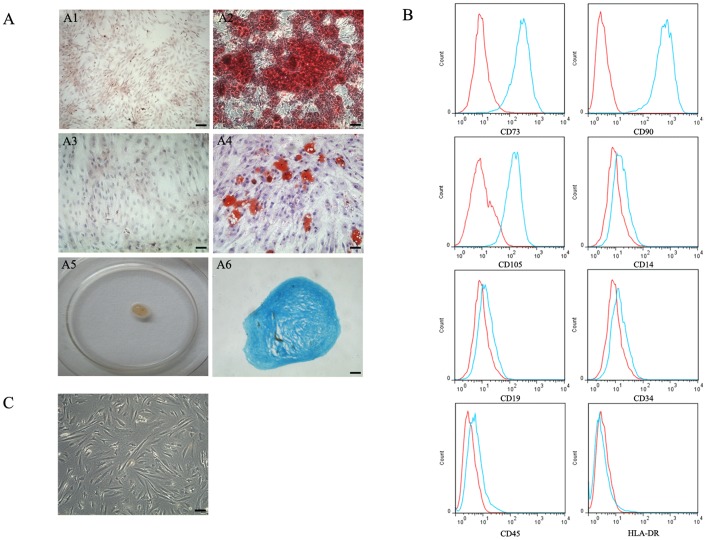
The Identification of CEP-derived stem cells (CESCs). A. In vitro trilineage differentiation assay of CESCs. CESCs were able to osteogenic, adipogenic and chondrogenic differentiation under appropriate conditions. The mineralized matrix, stained with alizarin red was shown in A2 after 21 days osteogenic differentiation, and no alizarin red positive matrix was found in cells cultured in growth medium (A1). The intracellular lipid droplets, stained with Oil Red O was shown in A4 after 21 days adipogenic differentiation, and no adipocytes were found in cells cultured in growth medium (A3). Cells cultured in chondrogenic induction medium formed an aggregate (A5), and its histologic section stained with alcian blue was shown in A6. B. flow cytometric immunophenotyping of CESCs. The green lines represent the fluorescence intensity of cells stained with the indicated antibodies and the red lines represent the negative control cells, which were stained with a non-immunoreactive isotype control antibody. C. Morphology of CESCs. They were spindle-shaped and polygonal-shaped adherent cells. Bar = 100 µm.

CESCs adhered to plastic and exhibited various morphologies that ranged from spindle-shaped to polygonal-shaped (Figure 2C). CESCs were immunophenotyped via flow cytometry in accordance with the immunophenotypic criteria of the International Society for Cellular Therapy [31]. Immunophenotypic evaluation demonstrated that CESCs were positive for CD73, CD90, and CD105, but negative for CD14, CD19, CD34, CD45 and HLA-DR (Figure 2B, Table 3). Most of these surface makers were consistent with the criteria of the International Society for Cellular Therapy, but the mean percentage of CD105 was slightly below the accepted level. The multipotency of CESCs was evaluated via differentiation into osteogenic (Figure 2 A1, A2), adipogenic (Figure 2 B1, B2) and chondrogenic (Figure 2 C1, C2) lineages over a period of 21 days, which indicated that CESCs were capable of differentiating into osteoblasts, adipocytes and chondroblasts under differentiating conditions *in vitro* tissue culture.

### Expression of MIF and CD74 in Human Degenerative IVD Tissues

**Figure 3:**
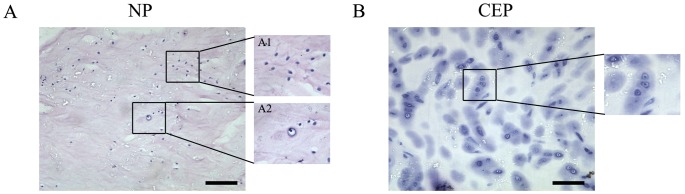
Histomorphology of degenerative human NP and CEP tissues. A. histologic morphology of NP with HE staining. Results for fibrocyte-like cells were shown in A1 and results for fibrochondrocyte-like cells were shown in A2. B. histologic morphology of CEP with HE staining. Bar = 100 µm.

NP cells were heterogeneous in their morphology and could be divided into two groups: cells in lacunae with an ovoid, fibrochondrocyte-like shape and cells with a spindle-shaped, fibrocyte-like morphology (Figure 3A1). A small percentage of cells were fibrochondrocyte-like in shape, and large fibrocyte-like cells were also observed (Figure 3A2). CEP cells were morphologically homogenous and chondrocyte-like cells were evident throughout CEP tissues (Figure 3B).

**Table 3:** Representative flow cytometry analysis.

Antigen	Percent positive	accepted mean percentage level
CD73	>95%	≥95%
CD90	>95%	≥95%
CD105	80–90%	≥95%
CD14	<2%	≤2%
CD19	<2%	≤2%
CD34	<2%	≤2%
CD45	<2%	≤2%
HLA-DR	<2%	≤2%

**Figure 4:**
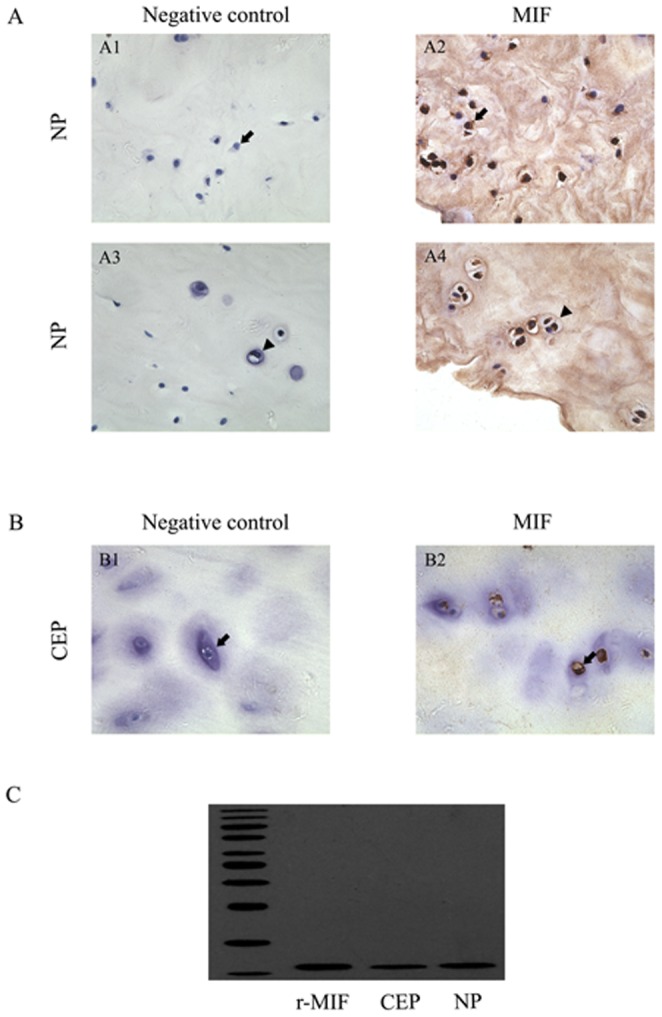
Immunohistochemistry staining for MIF in degenerative human NP and CEP tissues. A. Paraffin-embedded sections of human NP tissues. Results for negative controls were shown in A1, A3 and results for MIF-immunoreactive cells were shown in A2, A4. fibrocyte-like cells (arrow) were shown in A1 and A2, and fibrochondrocyte-like cells (arrowhead) were shown in A3 and A4. MIF-immunoreactive fibrocyte-like cells (arrow) were shown in A2 and MIF-immunoreactive fibrochondrocyte-like cells (arrowhead) were shown in A4. B. Paraffin-embedded sections of human CEP tissues. Results for immunoreactive-negative chondrocytes (arrow) were shown in B1 and results for MIF-immunoreactive chondrocytes (arrow) were shown in B2. Immunopositivity shows as brown staining and no immunopositivity was observed in negative controls. C. Degenerative IVD tissues from 3 human donors were homogenized and processed for western blot analysis with a rabbit polyclonal anti-human MIF antibody. A single protein band with estimated molecular masses of 12.5 kDa was observed. 20 ng of r-MIF was used as a standard. Bar = 50 µm.

MIF immunoreactivity was clearly identifiable in human degenerative IVD tissues (Figure 4A, B). The MIF-immunoreactive cells were distributed throughout the NP tissues (Figure 4A). MIF immunoreactivity was present on fibrocyte-like cells in the NP (arrow; Figure 4A2). MIF immunoreactivity was also found on fibrochondrocyte-like cells (arrowhead; Figure 4A4). In the CEP, MIF immunoreactivity was also found in chondrocyte-like cells (arrow; Figure 4B2). There was no immunoreactivity in negative control samples (Figure 4A1, A3 and B1). Western blotting for MIF indicated a protein band with an estimated molecular mass of 12.5 kDa; 20 ng of r-MIF was used as a standard (Figure 4C).

**Figure 5:**
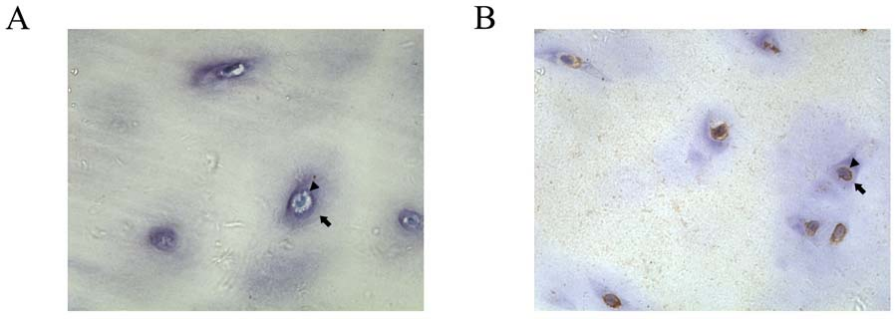
Immunohistochemistry staining for CD74 in degenerative human CEP tissues. Paraffin -embedded sections of human **CEP** tissues. Results for negative controls were shown in A and results for CD74-immunoreactive chondrocytes were shown in B. Immunopositivity shows as brown staining and no immunopositivity was observed in negative controls. The edge of the chondrocytes was marked by the arrowhead, and edge of the cartilage lacunae was marked by the arrow.

CD74 immunoreactivity was apparent in human degenerative CEP tissues (Figure 5). The CD74 immunoreactive cells were found on the membranes of chondrocyte-like cells (Figure 5B). There was no immunoreactivity in negative control samples (Figure 5A).

### TNF-α Upregulated MIF Expression in NP Cells

**Figure 6:**
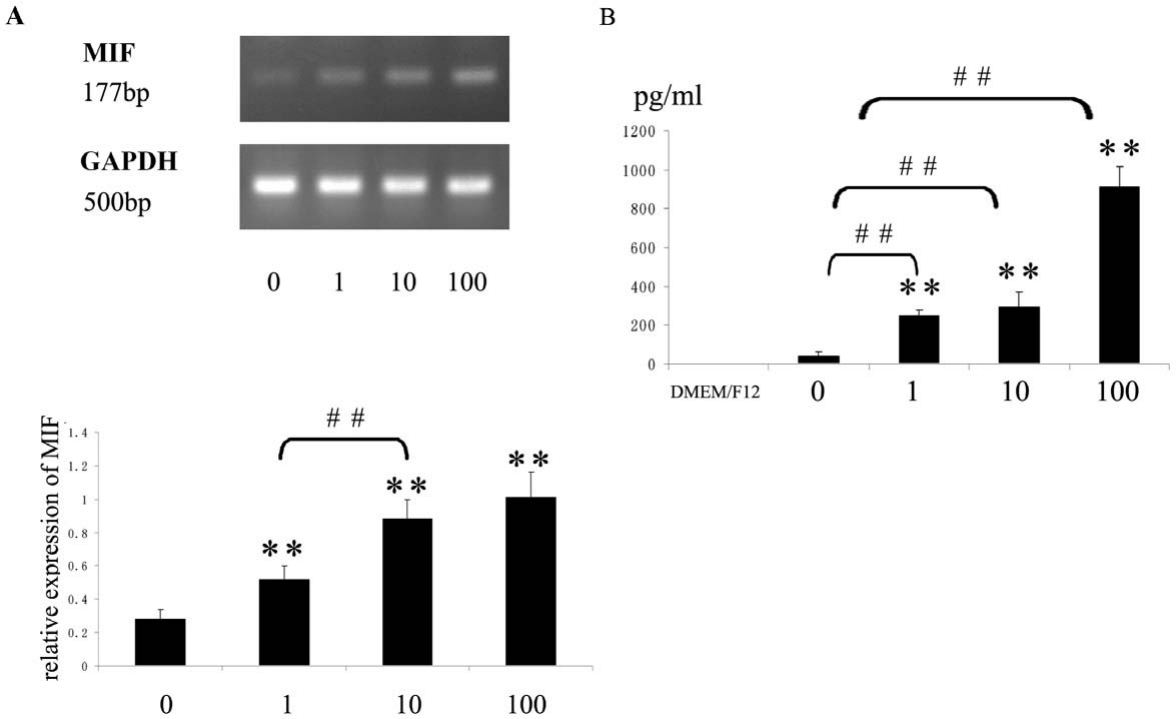
TNF-α increased mRNA and protein levels of MIF. A. TNF-α increased MIF mRNA levels in cultured human NP cells. NP cells were treated with various concentrations of TNF-α (0, 1, 10 and 100 ng/ml) for 6 h. total RNA was extracted and reverse-transcribed for RT-PCR. The expression level of MIF was normalized to GAPDH expression and the data were obtained from three independent experiment. **P<0.01 *vs* the corresponding control medium without stimuli. B. NP cells were treated with various concentrations of TNF-α (0, 1, 10 and 100 ng/ml) for 12 h. TNF-α increased MIF secretion in a dose-dependent pattern as analysed by ELISA. **P<0.01 *vs* DMEM/F12 group, and ##P<0.01 *vs* the corresponding control medium without stimuli. Statistical analysis data were expressed as means ± standard deviations.

TNF-α was added at different concentrations (0–100 ng/ml) to DMEM/F12 for 6 hours. NP cells expressed detectable levels of MIF mRNA in DMEM/F12 without TNF-α stimuli; however, only 1 ng/ml TNF-α increased the expression of MIF mRNA significantly, and TNF-α increased the expression of MIF mRNA in a dose-dependent pattern (Figure 6A). The expression of mRNA was standardized to GAPDH, as is shown in Figure 6A.

TNF-α was added at different concentrations (0–100 ng/ml) to DMEM/F12 for 12 hours. TNF-α also induced a dose-dependent increase of MIF in DMEM/F12 relative to DMEM/F12 alone, which was used as a negative control. No MIF was detected in DMEM/F12, and a small amount of MIF was secreted by NP cells in the absence of stimuli (Figure 6B). Following stimulation with TNF-α, MIF secretion was significantly increased (Figure 6B).

### The Effect of Human Degenerate NP Cells on the Migration of CESCs

**Figure 7:**
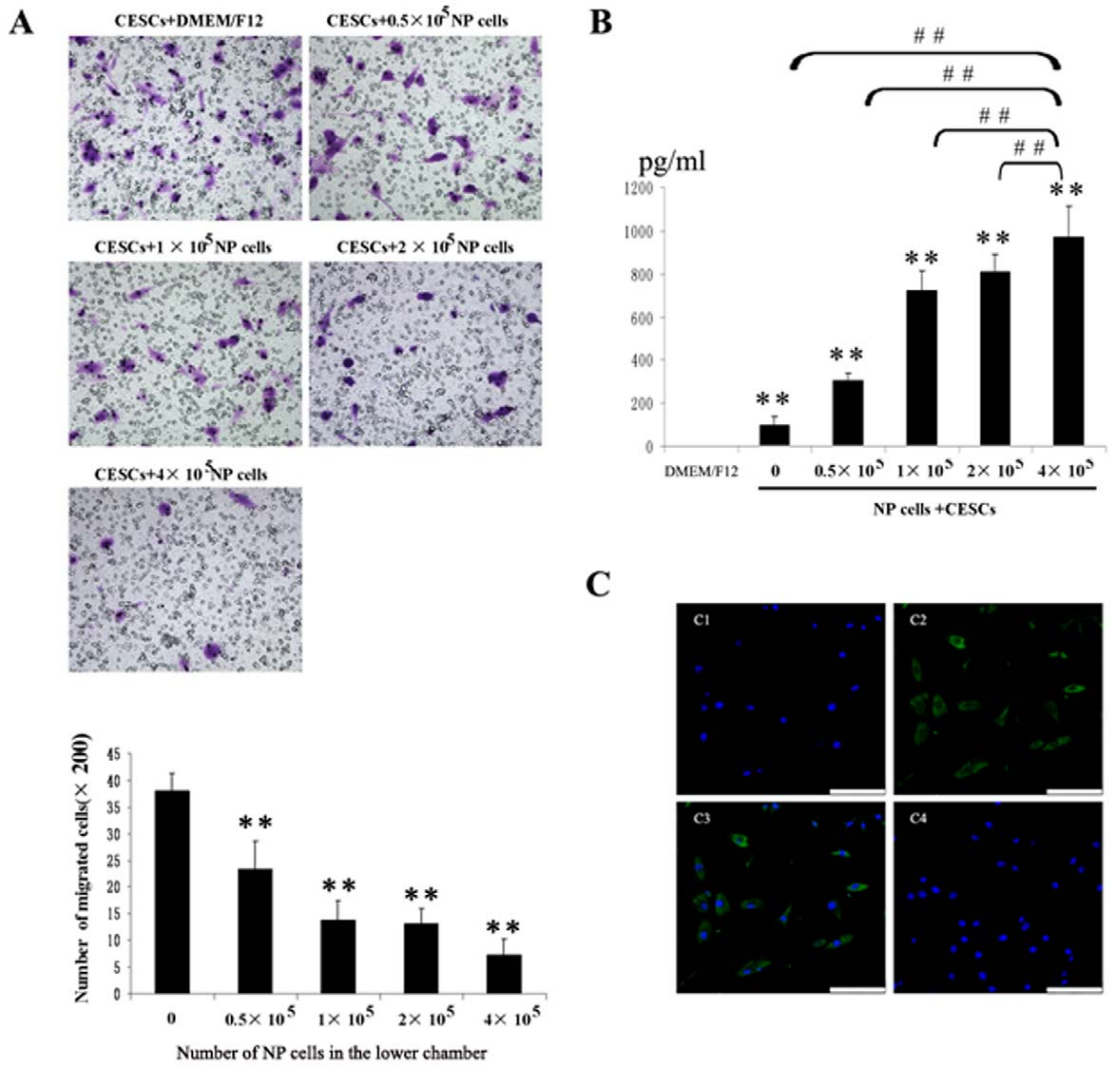
The effect of NP cells on the migration of CESCs. Inhibition of CESCs migration were examined by increasing the numbers of NP cells loaded in the lower chamber (from 0 to 4×10^5^). A. The membrane filter was stained with 1% crystal violet after migration assays. The numbers of NP cells loaded in the lower chamber were: 0, 0.5×10^5^, 1×10^5^, 2×10^5^, 4×10^5^. The mean numbers of migrated CESCs (mean standard ± deviation) were: 37.9±3.4, 23.3±5.3, 13.8±3.6, 13±3, 7.3±2.9(from 0 to 4×10^5^ ). **P<0.01 *vs* the corresponding control medium without stimuli. B. The CM was collected after migration assay and the MIF concentrations in the CM were measured by ELISA. **P<0.01 *vs* DMEM/F12 group, ##P<0.01 *vs* the corresponding control medium without stimuli. C. MIF immunoreactivity (green) was identified in cultured human NP cells. The merge image was shown in C3. No immunoreactivity was found in the negative controls (C4). Bar = 100 µm.

The mean numbers of migrated CESCs, with respect to the different numbers of NP cells, are shown in Figure 7A. CESCs were able to spontaneously migrate across transwell filters in DMEM/F12 without any interferon treatment. Relative to the control group, the mean numbers of migrated CESCs in the CM group decreased significantly from 37.9±3.4 cells/well to 7.3±2.9 cells/well. NP cells inhibited the migration of CESCs in a number-dependent manner.

The MIF value in the CM for the different co-culture systems are listed in Figure 7B. The concentrations of MIF in the CM increased significantly from 87.3 pg/ml to 1033.7 pg/ml. No MIF was detected in DMEM/F12, and a small amount of MIF was detected in the CM of CESCs.

### MIF Expression in Cultured Human Degenerate NP Cells

The expression of MIF in human degenerative NP cells was evaluated by immunohistological means. NP cells exhibited strong expression of MIF, and staining was observed in both the cell membrane and cytoplasm; however, no nuclear staining was observed (Figure 7C). No immunoreactivity was detected in the cell membrane or cytoplasm of IgG controls or when an anti-MIF antibody pre-absorbed with excess r-MIF was used (Figure 7C4).

### The Effects of NP Cell-derived MIF and ISO-1 on CESCs Migration

**Figure 8:**
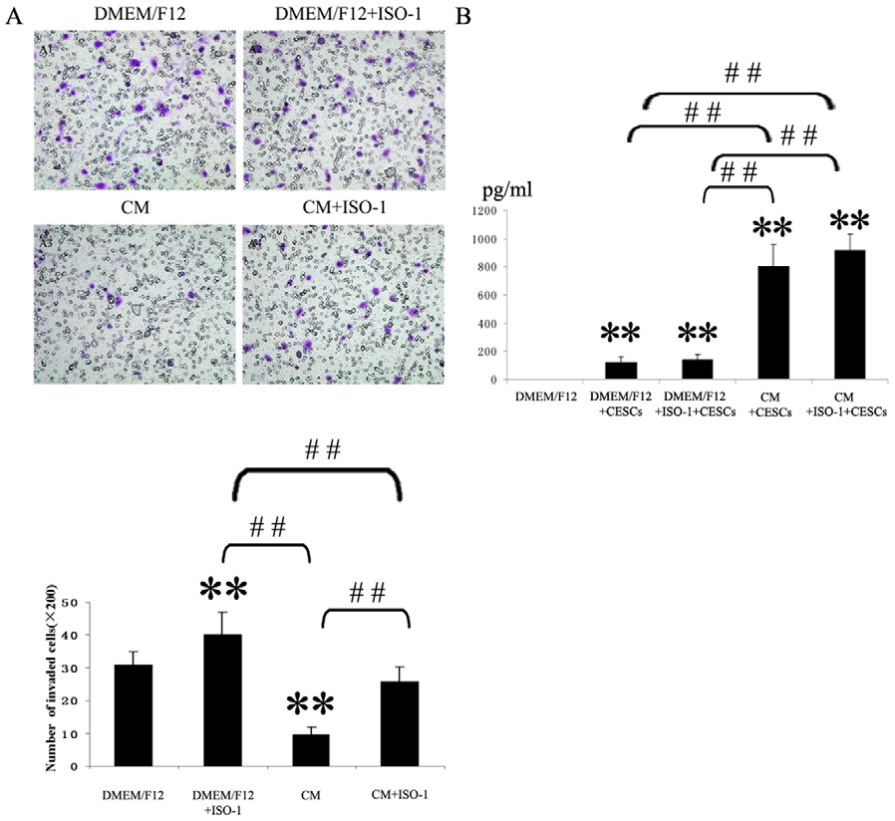
Effect of NP Cells-derived MIF and ISO-1 on CESCs migration. Restoration of CESCs migration were examined by co-incubation of ISO-1 with NP cells-derived MIF. A. The mean numbers of migrated CESCs (mean standard ± deviation) were: 30.8±4.4, 40.2±6.7, 9.5±2.6, 25.7±4.5 (from DMEM/F12 to CM+ISO-1). **P<0.01 *vs* DMEM/F12 group, and ##P<0.01. B. The CM was collected after migration assay and the MIF concentrations in the CM were measured by ELISA. **P<0.01 *vs* DMEM/F12 group, and ##P<0.01.

The mean number of migrated CESCs is shown for each condition in Figure 8A. Relative to the control group, the mean numbers of migrated CESCs in the CM group were significantly reduced from 30.8±4.3 cells/well to 9.5±2.6 cells/well. 10 µg/ml ISO-1 restored a significant amount of migration by inhibiting MIF, and the mean numbers of migrated CESCs in the CM+ISO-1 group increased from 9.5±2.6 cells/well to 25.7±4.5 cells/well. In particular, relative to the control group, the mean numbers of migrated CESCs in the DMEM/F12+ISO-1 group also increased significantly from 30.8±4.3 cells/well to 40.2±6.7 cells/well. There was no significant difference between the DMEM/F12 and CM+ISO-1 groups.

The MIF value in the CM group is shown for each condition in Figure 8B. The CM was obtained from the lower chamber after 24 hours of migration. The concentrations of MIF in the CM+CESCs and CM+CESCs+ISO-1 groups were increased significantly relative to the control group (P<0.01), while the concentrations of MIF in the DMEM/F12+CESCs and DMEM/F12+CESCs+ISO-1 groups were also significantly increased (P<0.05). The concentration of MIF in the CM+CESCs group was significantly increased relative to the DMEM/F12+CESCs group and was also increased in the CM+CESCs+ISO-1 group relative to the DMEM/F12+CESCs+ISO-1 group. However, there were no significant differences between the CM+CESCs and CM+CESCs+ISO-1 groups or between the DMEM/F12+CESCs and DMEM/F12+CESCs+ISO-1 groups. ISO-1 did not affect the expression of MIF in the CM group, and no MIF was detected in DMEM/F12.

### The Effect of r-MIF and ISO-1 on CESCs Migration

**Figure 9:**
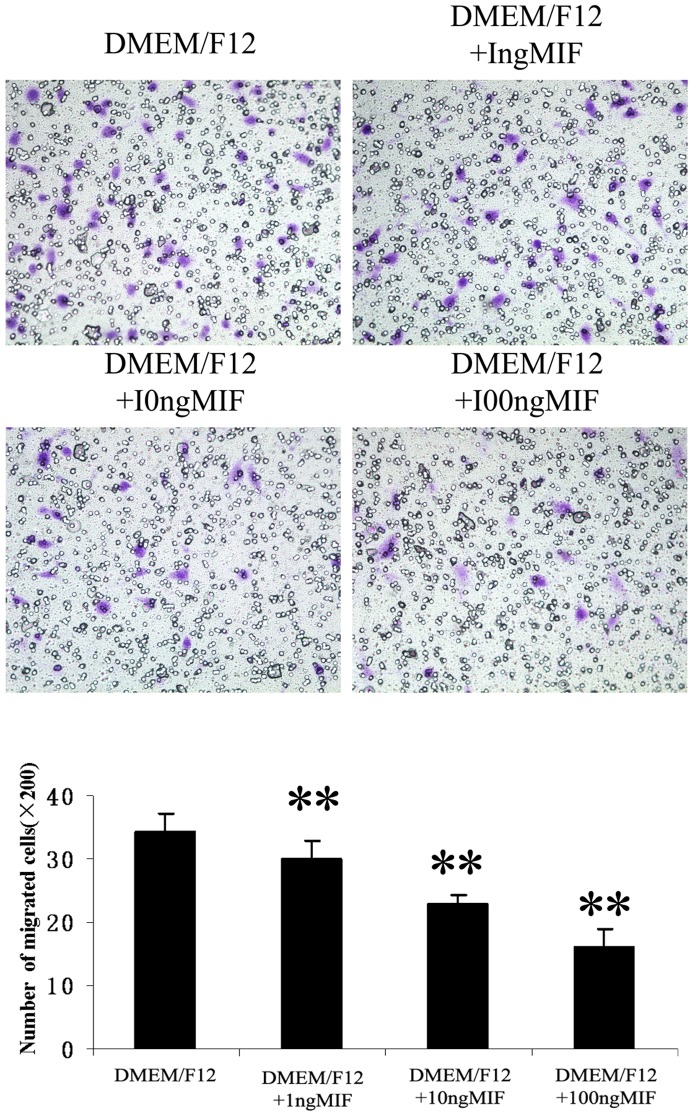
Effect of r-MIF on CESCs migration. The mean numbers of migrated CESCs (mean standard ± deviation) were: 34.2±2.9, 30±2.7, 22.7±1.4, 15.9±2.9 (from DMEM/F12 to DMEM/F12+100 ng MIF). **P<0.01 *vs* DMEM/F12 group.

**Figure 10:**
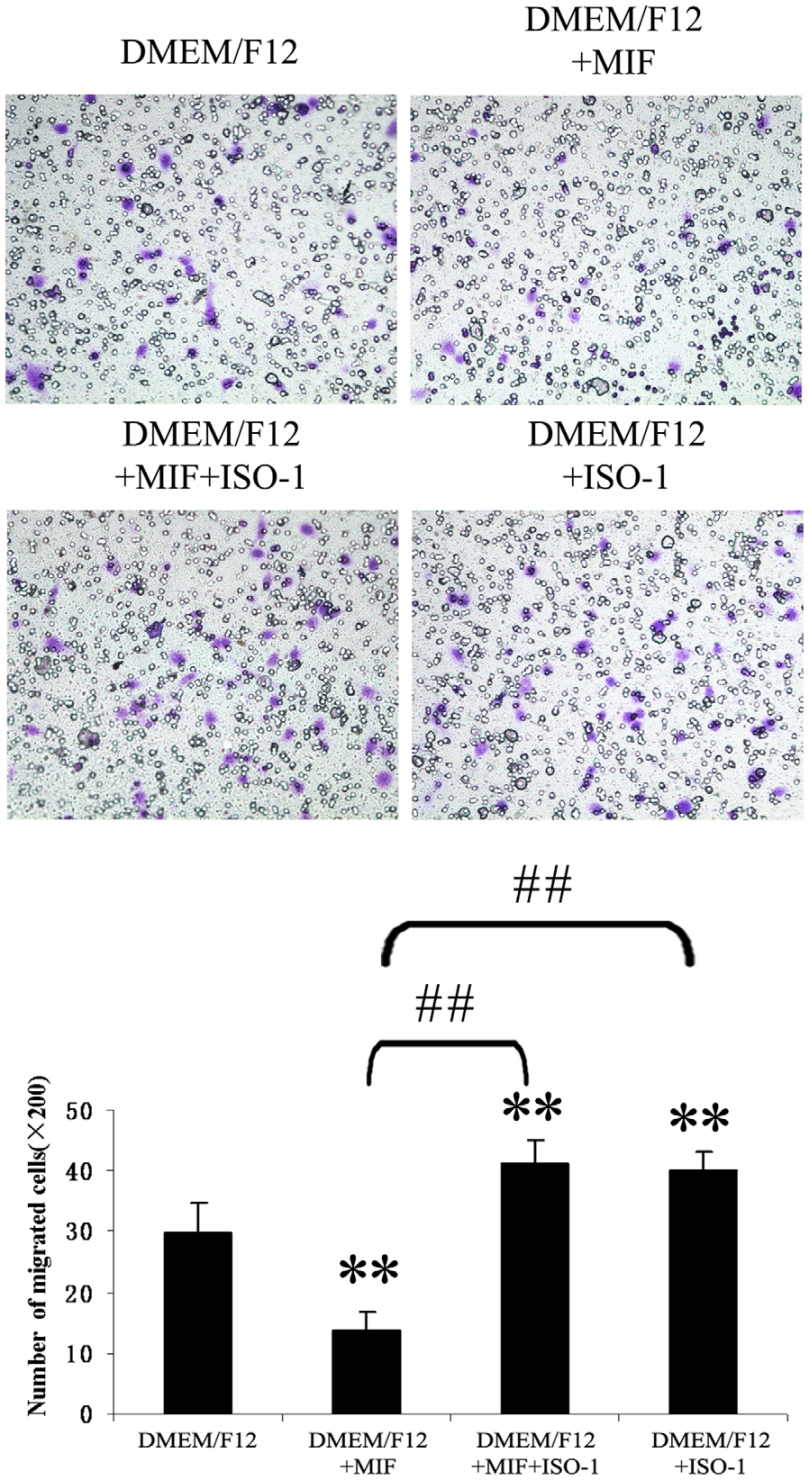
Effect of r-MIF and ISO-1 on CESCs migration. Restoration of CESCs migration were examined by co-incubation of ISO-1 with rMIF. The mean numbers of migrated CESCs (mean standard ± deviation) were: 29.8±5, 13.9±2.9, 41.4±3.8, 40.2±3.0 (from DMEM/F12 to DMEM/F12+ISO-1). **P<0.01 *vs* DMEM/F12 group, and ##P<0.01.

Different concentrations of r-MIF (1–100 ng/ml) added to the lower chamber prior to the start of the migration assay elicited dose-dependent inhibition of CESCs migration (Figure 9). When both r-MIF and ISO-1 were added to the lower chamber prior to the migration assay, CESCs migration was restored (Figure 10). Along with an increase in MIF concentration, the mean numbers of migrated CESCs were significantly decreased from 34.2±2.9 cells/well to 15.9±2.9 cells/well. Additionally, 100 µg/ml ISO-1 significantly restored migration by inhibiting 100 ng/ml r-MIF, which resulted in an increase from 13.9±2.9 cells/well to 41.4±3.7 cells/well. In particular, the mean numbers of migrated CESCs in both DMEM/F12+MIF+ISO-1 and DMEM/F12+ISO-1 groups were also significantly increased relative to the control group; however, there was no significant difference between the DMEM/F12+MIF+ISO-1 and DMEM/F12+ISO-1 groups.

### CD74Ab Inhibited the Migration of CESCs in vitro

**Figure 11:**
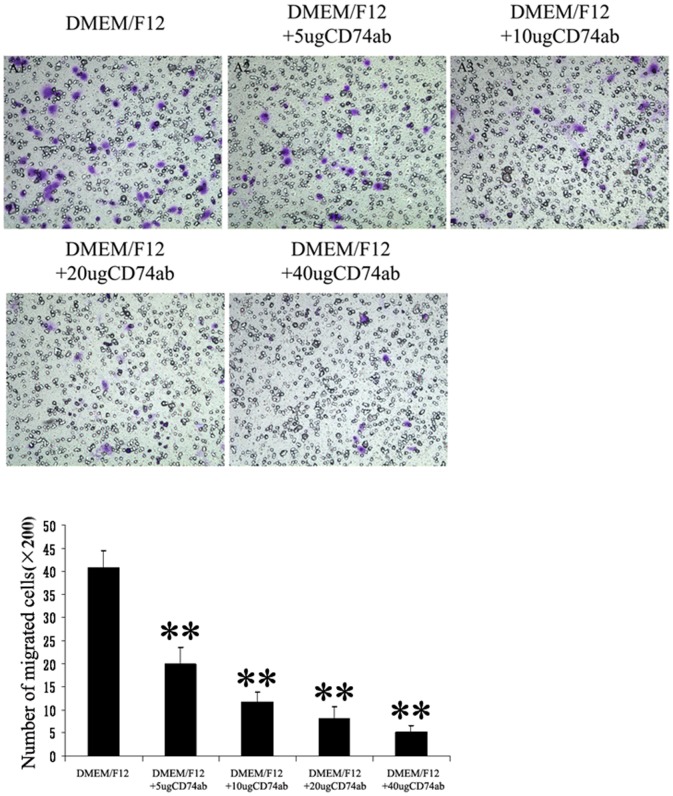
CD74Ab inhibited in vitro migration of CESCs. Inhibition of CESCs migration were examined by increasing concentrations (from 5 to 40 µg/ml) of CD74Ab in CM. The mean numbers of migrated CESCs (mean standard ± deviation) were: 40.8±3.4, 20±3.5, 11.7±2.1, 8±2.6, 5.1±1.5 (from DMEM/F12 to DMEM/F12+40 µg CD74Ab). **P<0.01 *vs* DMEM/F12 group.

Different concentrations of CD74Ab (5–40 µg/ml) have been used to test the effect of CD74 signaling on CESCs migration. The mean numbers of migrated CESCs are shown for each concentration of CD74Ab in Figure 11. CESCs were able to spontaneously migrate across transwell filters in the DMEM/F12 group without any interferon treatment. The CD74Ab inhibited CESCs migration in a dose-dependent manner. Along with an increase of CD74Ab concentrations, the mean numbers of migrated CESCs were significantly decreased from 40.8±3.5 cells/well to 5.2±1.5 cells/well. DMEM/F12 was used in the lower chamber as a control.

### CD74 Expression in Cultured CESCs

**Figure 12:**
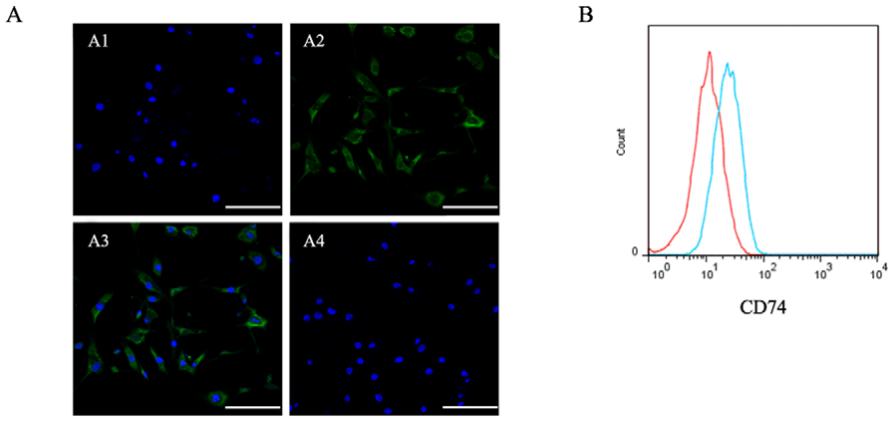
The MIF receptor CD74 on the CESCs. CD74 immunoreactivity (green) was identified in cultured human CESCs. The merge image was shown in A3. No immunoreactivity was found in the negative controls (A4). Bar = 100 µm. B. The green lines represent the fluorescence intensity of cells stained with the CD74 antibody and the red lines represent the negative control cells, which were stained with a non-immunoreactive isotype control antibody. The mean percentage of CD74 positive cells was between 30–40%.

The expression of CD74 in CESCs was evaluated by immunofluorescent analysis. CD74 immunoreactivity was clearly identifiable in CESCs in monolayer culture. A substantial proportion of the CD74 immunoreactivity was found in both membrane and cytoplasm of those CESCs; however, no nuclear staining was observed (Figure 12A). No immunoreactivity was detected in the cell membrane or in the cytoplasm of IgG controls (Figure 12A4). Furthermore, cell surface expression of CD74 was evident in the CESCs (Figure 12B).

### CD74 siRNA on the Migration of CESCs

**Figure 13:**
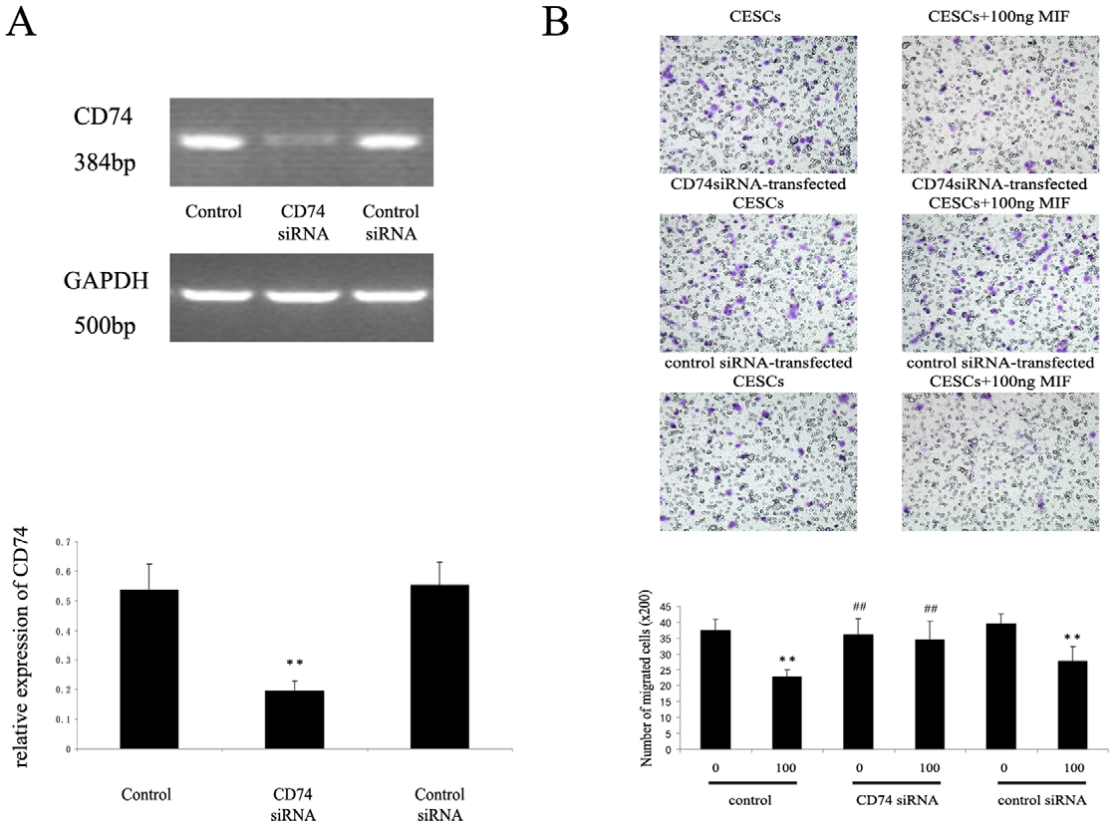
Detection of CD74 and effect of CD74 knockdown on the migration of CESCs. CESCs were transfected with CD74 siRNA or negative control siRNA and then stimulated with 100 ng/ml MIF for 24 h. A. CD74 siRNA significantly inhibited CD74 mRNA expression. **P<0.01 vs the corresponding control medium or negative control siRNA. B. The migration of CESCs in the CD74 siRNA group was not significantly decreased in response to 100 ng/ml MIF. However, the migration of CESCs in the control group or siRNA control group was significantly decreased in response to 100 ng/ml MIF. **P<0.01 vs the corresponding control medium without stimuli, and ##P<0.01 vs MIF-stimulated cells without CD74 siRNA treatment.

To assess whether the observed inhibitory effects of r-MIF on the migration of CESCs were mediated by CD74, CD74 expression in CESCs was knocked down using specific CD74 siRNA. CD74 mRNA expression was significantly inhibited in the CD74 siRNA group compared with that in the control group or control siRNA group (Figure 13A). Our data showed that CD74 siRNA significantly decreased the inhibitory effects of r-MIF on the migration of CESCs. The migration of CESCs treated with CD74 siRNA was significantly restored in response to 100 ng/ml r-MIF, and the migration of CESCs in the control group or control siRNA group was still inhibited by 100 ng/ml r-MIF. However, there is no significant difference between 0 ng/ml MIF group and 100 ng/ml MIF group in the CD74 siRNA group(Figure 13B).

## Discussion

Minogue et al demonstrated that MIF mRNA was more highly expressed in NP cells than in articular cartilage (AC) and annular fibrosus (AF) cells [33]. However, the potential role of MIF in IVD degeneration had not yet been investigated. Bonnie et al reported that MIF can activate CD74 on human MSCs and inhibit their migration. The capacity of MSCs to home to injured tissues might be impaired by expression of MIF [14], [15]. We have now demonstrated that MIF is present in degenerate NP and CEP. Because MIF was also highly expressed in patients with autoimmune diseases and inflammation, those patients were excluded from the current study [1]–[3]. Similar to the aforementioned study by Bonnie L, we have now shown that MIF inhibits the migration of CESCs by reacting with the CD74 receptor. To our knowledge, this is the first report to clarify a regulatory mechanism governing CESCs migration in degenerated IVD.

Many adult tissues contain a population of MSCs that have the ability to renew following trauma or disease, or in response to aging. MSCs are capable of homing to injured or degenerated tissues where they exert therapeutic effects by providing cytokines and chemokines and enhancing both proliferation and differentiation of resident stem cells [12]. MSCs in human degenerate IVD tissues were first discovered by Risbud in 2007; since that time, a series of attempts have been made to characterize these cells [34]–[37]. IVD-derived MSCs exhibit biological characteristics similar to those of BM-MSCs [34]. Subsequently, we successfully isolated and characterized CESCs [26], [27] and found that they could be classified as MSCs according to the criteria established by the International Society for Cellular Therapy (ISCT) [31]. Moreover, in comparisons with BM-MSCs from the same subjects, CESCs have shown greater potential for chondrogenic and osteogenic differentiation than BM-MSCs [26], [27]. However, up to now, there is no study to identify whether IVD-derived MSCs migration exist or clarify the cell migration regulatory mechanism.

MIF is an important multifunctional cytokine involved in many physiopathologic processes, such as rheumatoid arthritis, inflammatory bowel disease and septic shock [1]–[3]. All of these conditions are associated with an inflammatory response in the affected organ; this is also true in the case of IVD degeneration [5]–[9]. Moreover, MIF is a potent inducer of a variety of pro-inflammatory cytokines expressed in degenerate IVD tissues, such as TNF-α, IL- 1β, IL-2, IL-6, IL-8, and IFN-γ[1]–[3]. MIF can also upregulate metalloproteinases (MMPs), which are associated with IVD degradation and resorption [38]. So MIF might promote IVD degeneration and induce discogenic pain by activating other effector cells via release of proinflammatory cytokines and MMPs. However, the exact mechanism remains to be elucidated.

Kim et al has demonstrated that CEP chondrocytes are capable of migration, which is stimulated by soluble factors produced by notochordal cells. CEP chondrocytes possess motile properties, such as cytoskeletal arrangement and filopodia, and their migration is regulated by a variety of molecules, including growth factors, matrix proteins and inflammatory cytokines [23]. They found that with the migration of CEP chondrocytes into the NP site notochordal NP gradually transited into fibrocartilaginous NP [21]. Several studies proved that inflammatory responses occur in degenerate NP tissues, and a variety of proinflammatory or inflammatory cytokines are involved in IVD degeneration [5]–[9]. In our hypothesis, the migration of CESCs into the NP site aims to exert regenerative effects, whereas, inflammated NP local micro-environment might induce migrated CESCs differentiating into fibrocytes, and further accelerate degenerative process. Thus inhibition of MIF might play a therapeutic role for IVD degeneration by promoting the migration of CESCs into the NP, and on the other hand, relieving MIF-induced inflammatory response in the NP site.

This study demonstrates that NP-secreted MIF inhibits CESCs migration. Recent studies have shown that degenerate human IVD tissues spontaneously secrete a variety of proinflammatory cytokines and can respond to noxious stimuli by increasing their production of proinflammatory cytokines. In addition to inflammatory cells in the degenerate IVD, NP cells are the main source of proinflammatory cytokines [24], [25], [39]. Proinflammatory mediators, such as TNF-α, are significantly increased during IVD degeneration and are potent inducers of MIF synthesis in macrophages and endometrial stromal cells [30], [32], [40]. NP cells secreted significant amounts of MIF after stimulation with relatively low concentrations of TNF-α (*i.e.*, 1 ng/ml), which occurred in a dose-dependent fashion (Fig. 6). NP cells derived from degenerate human IVD tissues had already experienced a TNF-α stimulus prior to tissue procurement, which might be the reason why they secreted a certain amount of MIF without any exogenous stimuli (Fig. 6). Determining whether other proinflammatory cytokines can increase MIF expression during IVD degeneration will require further study.

Kim et al has demonstrated that notochordal cells stimulate migration of CEP chondrocytes in a number-dependent manner, whereas, we found that NP cells could inhibit the migration of CESCs also in a number-dependent manner. The results of ELISA assays revealed that MIF levels in the CM by NP cells increased significantly; this corresponded to the increased number of NP cells in the lower chamber (Fig. 7B). Similar to the findings of Kim, cell migration assays indicated that CESCs are capable of spontaneous migration (Fig. 7A), and CESCs also secrete a certain amount of MIF (Fig. 8B). In Fig. 7A, when 10 µg ISO-1, a specific antagonist of MIF [14]–[17], was added into the DMEM/F12 medium to inhibit MIF biological activities, migration of CESCs was significantly restored (DMEM/F12+ ISO-1 group vs DMEM/F12 group). So it can be inferred that CESCs may, upon stimulation, secrete MIF to inhibit the migration of themselves by autocrine or paracrine pattern. In Fig. 8B, there were no significant differences in MIF levels between the DMEM/F12+CESCs and DMEM/F12+CESCs+ISO-1 groups, which indicated that ISO-1 did not affect the expression of MIF in CM. In Fig. 8A, when comparing the DMEM/F12+ ISO-1 group with the CM + ISO-1group, ISO-1 could only partially restore the migratory ability of CESCs in the CM, This suggests that the CM may contain some other cytokines, in addition to MIF, which also can suppress CESCs migration, and that the biological activities of those cytokines cannot be inhibited by ISO-1.

CD74 is an integral membrane protein and the major histocompatibility complex (MHC) class II-associated invariant chain, which is located in the plasma membrane. CD74 regulates the functions of MHC class II molecules and also acts as a receptor for MIF; thus, many biological activities of MIF are mediated by CD74 [41]. Recent studies have shown that CD74 is expressed on MSCs and that the activation of CD74 inhibits the migration of human MSCs [14], [15]. In support of these findings, we have demonstrated that CD74 is expressed by CESCs (Fig. 4, Fig. 11) and that the interaction of MIF and CD74 inhibits the migration of CESCs (Fig. 10). MIF-CD74 signal transduction could constitute a mechanism by which CESCs migration is regulated in degenerate IVD, which in turn can suppress the homing of CESCs to sites of inflammation. The restoration of migration with a specific inhibitor in the signal way might facilitate IVD repair and regeneration.

Recently, several types of MSCs from various compositions were found in degenerative IVD tissues [26], [27], [34], [37]. In situ stimulation of endogenous MSCs in IVD tissue seems to be an effective means to repair and regenerate degenerate IVDs. Moreover, MSCs from the same tissue may have a developmental repertoire that closely resembles that of IVD cells, which could facilitate the repair of IVD degeneration. Proinflammatory cytokines in degenerate IVD tissues, such as MIF, may interfere with the homing of MSCs. Our findings support the notion that MIF expressed in IVD may interfere with the capability of CESCs to home to sites of inflammation following tissue injury or degeneration. Inhibiting the function of MIF, and restoring the migration of CESCs, might promote IVD repair and regeneration. So ISO-1 might be a useful new therapeutic tool in the treatment of IVD degeneration. However, the exact role of MIF and migration of CESCs in vivo remains largely unknown; therefore, further experiments will be needed.
