# Correction: Macrophage Migration Inhibitory Factor Inhibits the Migration of Cartilage End Plate-Derived Stem Cells by Reacting with CD74

**DOI:** 10.1371/annotation/a5edef40-e46d-4810-9008-dbda429ccc2c

**Published:** 2012-11-09

**Authors:** Cheng-jie Xiong, Bo Huang, Yue Zhou, Yan-ping Cun, Lan-tao Liu, Jian Wang, Chang-qing Li, Yong Pan, Hai Wang

The first two authors, Cheng-jie Xiong and Bo Huang, should be noted as contributing equally to this work.

Author Bo Huang should also be listed as a corresponding author, and can be contacted at the following email address: fmmuhb@126.com

The following information was missing from the Funding section: This work was also supported by the National Natural Science Foundation of China (81101364 and 81071498). 

